# PTPRB promotes metastasis of colorectal carcinoma via inducing epithelial-mesenchymal transition

**DOI:** 10.1038/s41419-019-1554-9

**Published:** 2019-04-30

**Authors:** Xingyue Weng, Wei Chen, Wangxiong Hu, Kailun Xu, Lina Qi, Jiani Chen, Demin Lu, Yinkuan Shao, Xi Zheng, Chenyang Ye, Shu Zheng

**Affiliations:** 1 Cancer Institute (Key Laboratory of Cancer Prevention and Intervention, China National Ministry of Education), The Second Affiliated Hospital, Zhejiang University School of Medicine, Hangzhou, Zhejiang 310009 China; 2 Cancer Institute of Integrated Traditional Chinese and Western Medicine, Zhejiang Academy of Traditional Chinese Medicine, Tongde Hospital of Zhejiang Province, Hangzhou, Zhejiang 310012 China

**Keywords:** Cancer, Cell biology

## Abstract

Dysregulation of protein tyrosine phosphatase, receptor type B (PTPRB) correlates with the development of a variety of tumors. Here we show that PTPRB promotes metastasis of colorectal cancer (CRC) cells via inducing epithelial-mesenchymal transition (EMT). We find that PTPRB is expressed at significantly higher levels in CRC tissues compared to adjacent nontumor tissues and in CRC cell lines with high invasion. PTPRB knockdown decreased the number of invasive CRC cells in an in vitro wound healing model, and also reduced tumor metastasis in vivo. Conversely, PTPRB overexpression promoted CRC cell invasion in vitro and metastasis in vivo. PTPRB overexpression decreased vimentin expression and promoted E-cadherin expression, consistent with promotion of EMT, while PTPRB knockdown had the opposite effect. Hypoxic conditions induced EMT and promoted invasion in CRC cells, but these effects were eliminated by PTPRB knockdown. EMT blockade via TWIST1 knockdown inhibited the migration and invasiveness of CRC cells, and even increased PTPRB expression could not reverse this effect. Altogether, these data support the conclusion that PTPRB promotes invasion and metastasis of CRC cells via inducing EMT, and that PTPRB would be a novel therapeutic target for the treatment of CRC.

## Introduction

Colorectal cancer (CRC) is one of the most common cancers, and is diagnosed in more than 1 million patients each year^1^. Moreover, CRC is the fourth most common cause of cancer-related deaths after lung, liver, and stomach cancer^2^. Surgical resection has been considered as the most effective treatment for patients with CRC. However, despite significant advances in perioperative management and expansion of screening programs, CRC mortality continues to increase rapidly worldwide^3^. Metastasis and recurrence are believed to be responsible for limiting long-term survival of patients with CRC, and there is an estimated recurrence rate of 29–63% among patients with stage II–III CRC^4^. According to previously reported data, the long-term survival of CRC patients with liver metastasis is rarely longer than three years^5^. Therefore, further understanding the vital mechanisms underlying CRC progression is urgent for developing new therapeutic strategies to improve prognosis.

Protein tyrosine phosphatase, receptor type B (PTPRB), also known as VE-PTP and RPTPβ, is located on chromosome 12q15^6^. The chromosome 12q15 locus contains multiple proliferation-related genes, and patients with deletions of the chromosome 12q15 region commonly present with global developmental delay and growth retardation^7^. PTPRB belongs to the protein tyrosine phosphatase family, and consists of an extracellular domain with multiple fibronectin type III-like domains, a single intracellular catalytic domain with C-terminal phosphorylation sites, and a transmembrane domain. Multiple studies have previously demonstrated that PTPRB plays critical roles in regulating various biological processes depending on binding and dephosphorylation of many types of receptor tyrosine kinases (RKTs)^8^. Soady et al. showed that PTPRB negatively regulates branching morphogenesis in the mammary epithelium, dependent on inhibition of FGFR activation and ERK1/2 phosphorylation^9^. Genetic studies in both mammals and invertebrate model systems have shown that the RPTP family is essential for tubular organ development^10^.

Moreover, dysregulation of PTPRB function and expression has been shown to correlate with carcinogenesis and tumor progression in multiple cancer types^11–13^. Qi et al. reported that PTPRB could decrease the level of Src phosphorylation, resulting in reduced cell proliferation and inhibitory tumorigenesis in non-small cell lung carcinoma^14^. Activation of the insulin signaling pathway is a very common phenomenon in many cancers^15^. PTPRB preferentially dephosphorylates the insulin receptor at Y960 and Y1146, suppressing insulin-induced activation of the insulin receptor and Akt^16^.

In this study, we used CRC cell lines to explore the expression of PTPRB and to analyze the biological function of PTPRB protein, with a focus on invasion and epithelial-mesenchymal transition (EMT). The results confirmed that PTPRB is highly expressed in CRC tissues and CRC cell lines with high invasion, and that PTPRB knockdown suppresses CRC cell migration and invasion in vitro and metastasis in vivo dependent on inhibition of EMT.

## Results

### PTPRB is associated with the motility and invasiveness of CRC cells

**Fig. 1:**
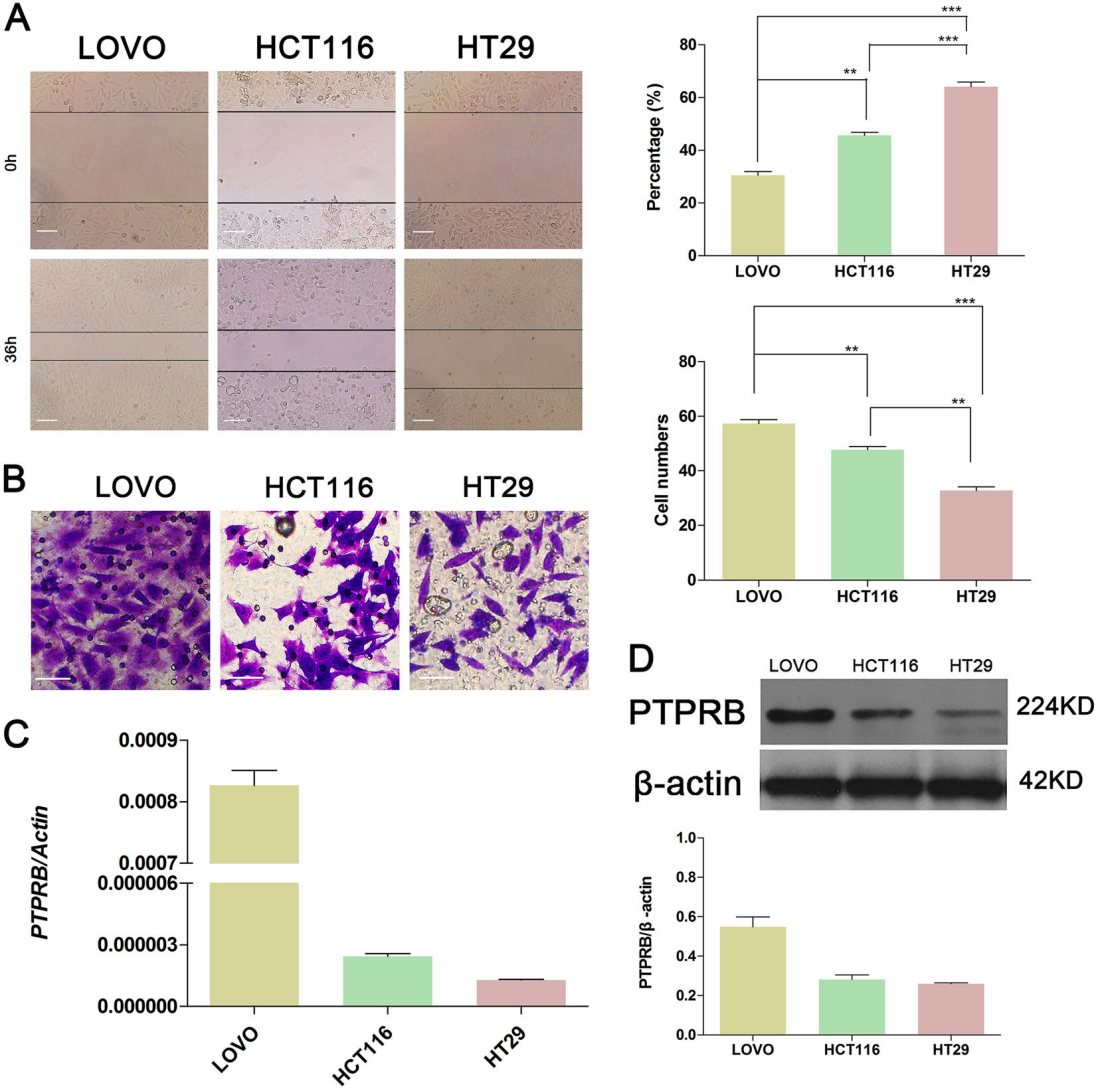
**a** The wound recovery ratio after incubation for 36 h for LOVO, HCT116, and HT29 cells, **P < 0.01, ***P < 0.001. **b** The number of invasive cells in LOVO, HCT116, and HT29 lines, **P < 0.01, ***P < 0.001. **c**, **d** The expression level of PTPRB in LOVO, HCT116, and HT29 cells was detected at the transcript level by RT-PCR and protein level by western blotting analysis

The motility and invasiveness of three CRC cell lines (LOVO, HCT116, and HT29) were analyzed by the transwell assay and wound healing assay. After 36 h incubation, the wound distance was reduced by 70% in LOVO, 50% in HCT116, and 30% in HT29 cells (Fig. 1a). Similarly, the frequency of invasiveness was the highest in LOVO, intermediate in HCT116, and lowest in HT29 cells (Fig. 1b). Interestingly, the expression of PTPRB at both the transcriptional level (Fig. 1c) and protein level (Fig. 1d) was high in LOVO, intermediate in HCT116, and low in HT29 cells. These results indicate that the expression of PTPRB in cells with different motility rates was different. The expression of PTPRB in cells with high motility rate was higher.

### PTPRB promotes migration and invasion in CRC cell lines

**Fig. 2:**
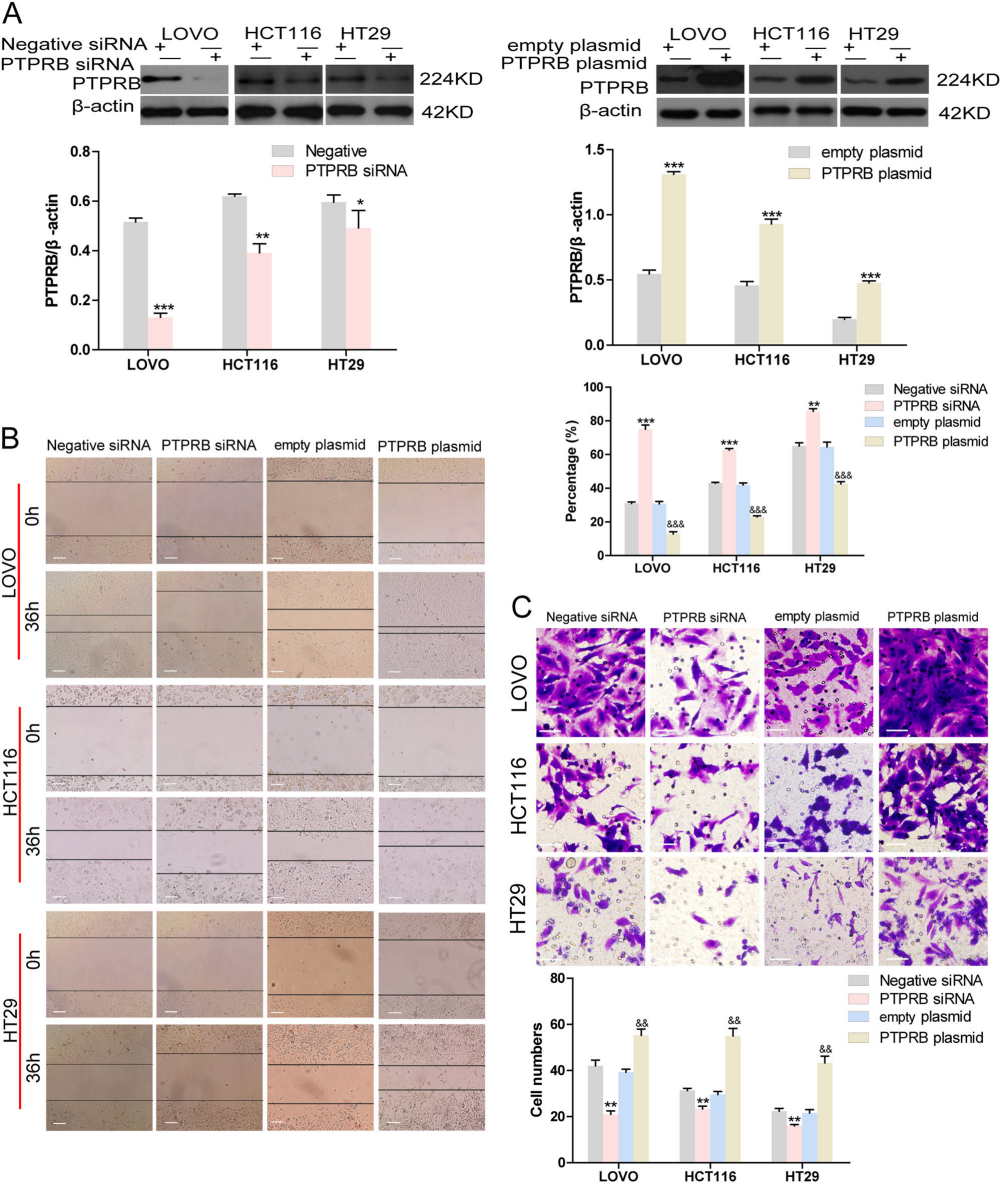
**a** The expression level of PTPRB in LOVO, HCT116, and HT29 cells after transfection of PTPRB-siRNA or PTPRB, *P < 0.05,**P < 0.01, ***P < 0.001. **b**, **c** The wound recovery ratio and number of invasive cells in LOVO, HCT116, and HT29 cells transfected with scrambled-siRNA, PTPRB-siRNA, or PTPRB plasmid, *P < 0.05,**P < 0.01, ***P < 0.001

siRNAs targeting PTPRB and PTPRB overexpression plasmids were, respectively, used to decrease and upregulate PTPRB expression in three CRC cell lines. Western blotting showed the transfection efficiency of PTPRB siRNA or PTPRB plasmid in all cell lines (Fig. 2a). The PTPRB-siRNA-transfected CRC cells showed a significant decrease in motility compared to the negative siRNA. Conversely, PTPRB overexpression accelerated the rate of wound healing in the CRC cell lines (Fig. 2b). A similar effect of PTPRB on invasiveness was observed. PTPRB knockdown significantly decreased the number of invasive cells compared to negative siRNA, while PTPRB overexpression promoted invasiveness (Fig. 2c).

### PTPRB regulates EMT in CRC cell lines

**Fig. 3:**
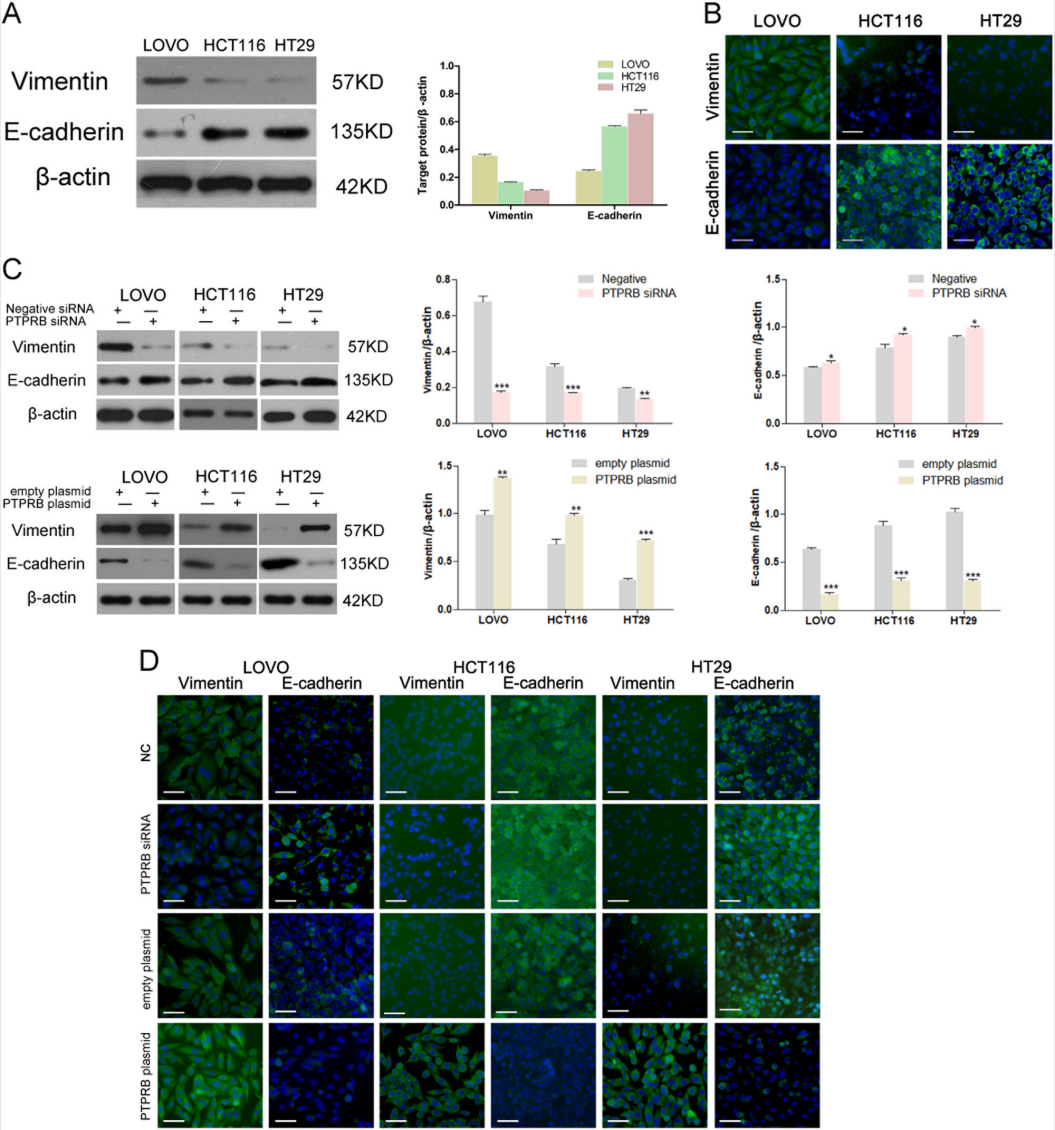
**a**, **b** Vimentin and E-cadherin expression was detected in LOVO, HCT116, and HT29 cells by western blotting analysis and immunofluorescence analysis. **c**, **d** Vimentin and E-cadherin expression in LOVO, HCT116, and HT29 cells transfected with PTPRB-siRNA or PTPRB plasmid was analyzed by western blotting analysis, *P < 0.05, **P < 0.01, ***P < 0.001 and immunofluorescence analysis

**Fig. 4:**
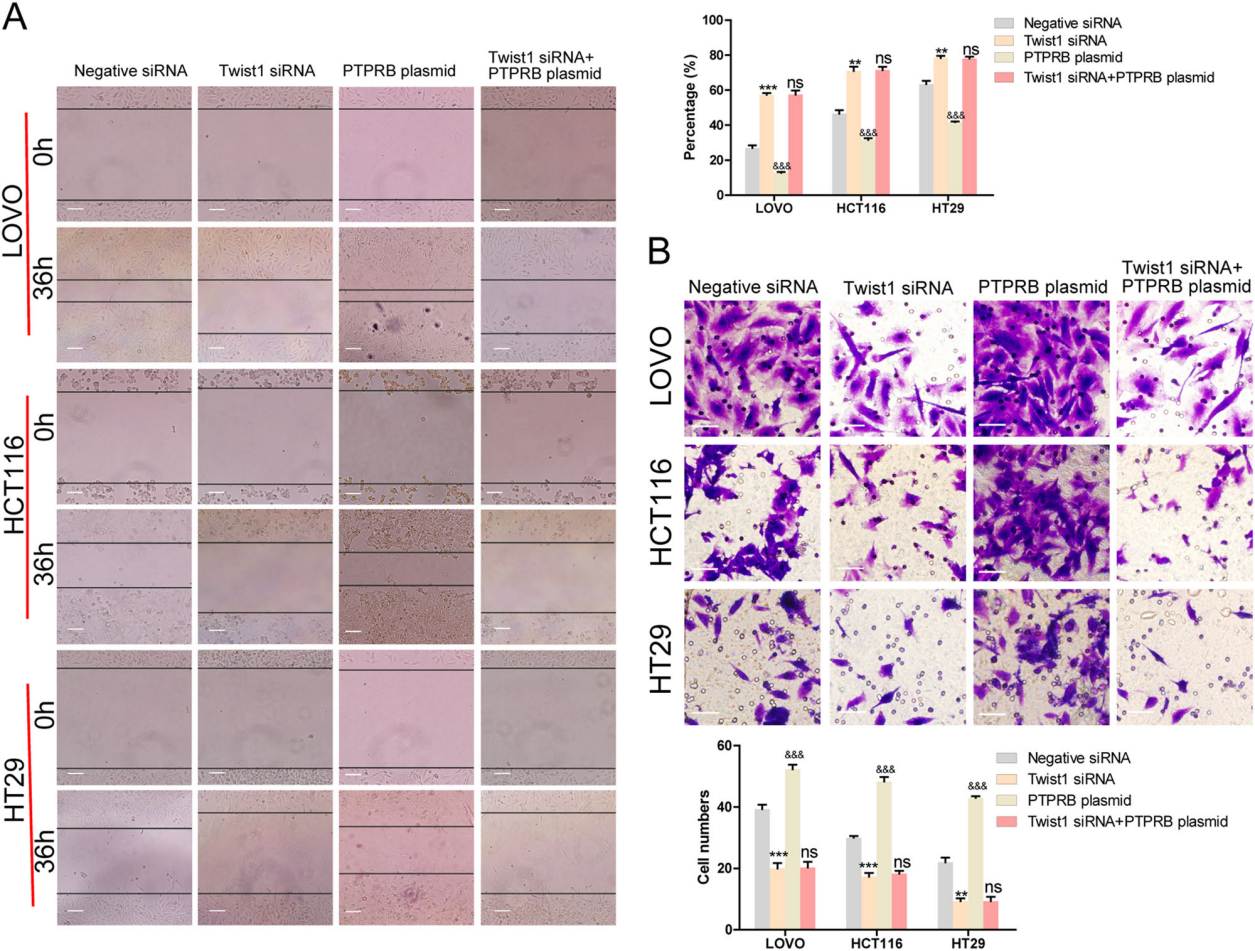
**a**, **b** The wound recovery ratio and number of invasive cells in LOVO, HCT116, and HT29 cells transfected with negative-siRNA, Twist1-siRNA, PTPRB plasmid, or Twist1-siRNA + PTPRB plasmid, **P < 0.01, ***P < 0.001

The expression level of vimentin ranged from high to low in LOVO, HCT116, and HT29 cells, and an opposite trend in expression was observed for E-cadherin (Fig. 3a, b). This result suggested that EMT was essential for CRC cell migration and invasion. We therefore investigated the relationship between PTPRB expression and the EMT process in CRC cells. After decreasing PTPRB expression, CRC cells showed reduced vimentin expression and increased E-cadherin expression. On the contrary, PTPRB overexpression promoted vimentin expression and downregulated E-cadherin expression (Fig. 3c). In line with the results of western bolt, PTPRB siRNA could decrease the fluorescence of vimentin and increased the fluorescence of E-cadherin, while PTPRB overexpression had the opposite effect (Fig. 3d). At the same time, PTPRB siRNA could reduce twist expression while PTPRB overexpression increased twist expression (Fig. S). Finally, a siRNA-targeting Twist1 was transfected into CRC cells to inhibit the EMT process. As anticipated, Twist1 knockdown significantly inhibited CRC cell migration and invasion (Fig. 4a, b). In addition, Twist1 knockdown also eliminated the effect of PTPRB overexpression on promoting CRC cells invasion.

### PTPRB knockdown inhibits hypoxia-induced metastasis in CRC cells

**Fig. 5:**
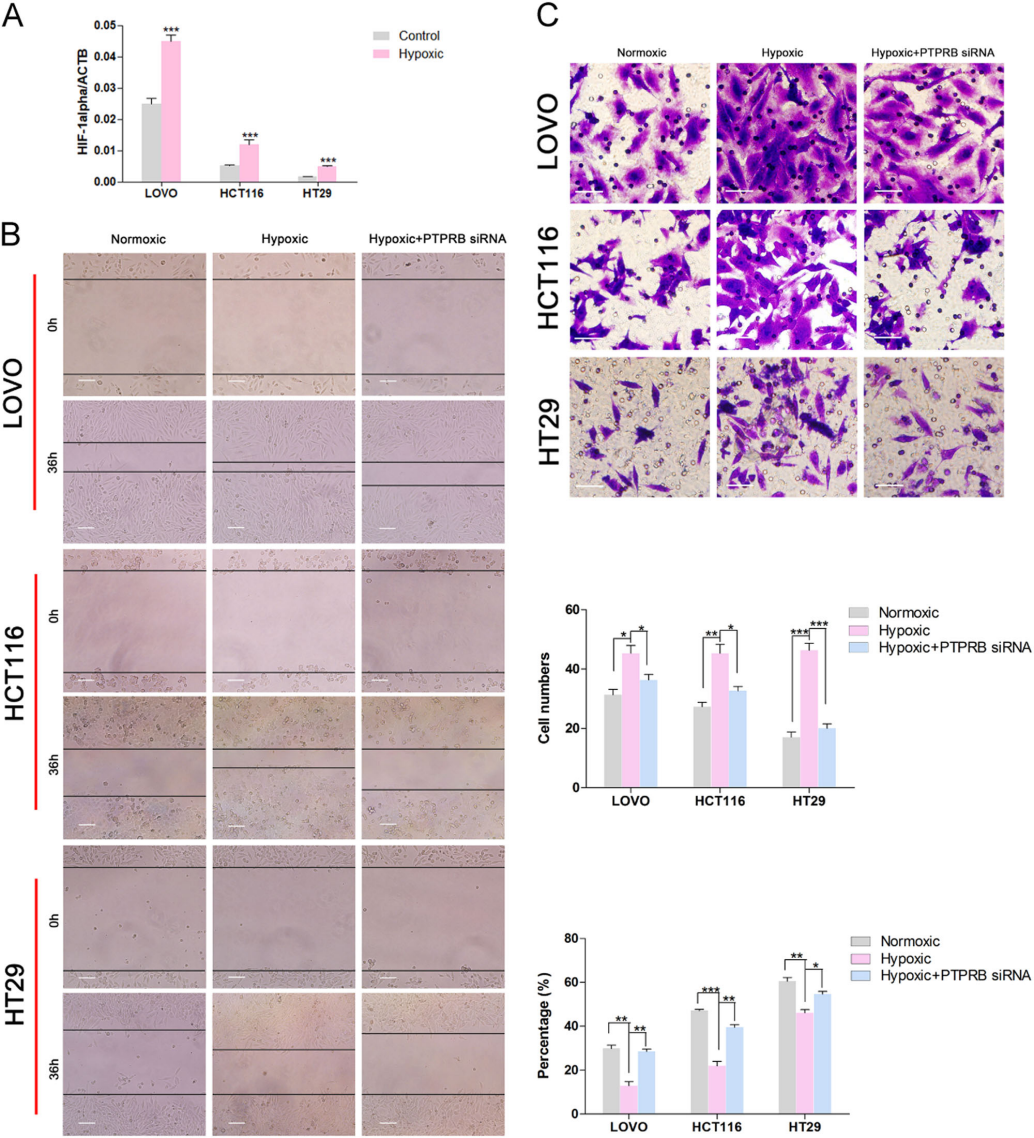
**a** The expression level of Hif-1α in LOVO, HCT116, and HT29 cells was detected under hypoxic condition by RT-PCR. **b**, **c**The wound recovery ratio and number of invasive cells in LOVO, HCT116, and HT29 cells after incubation in normoxic conditions, hypoxic conditions, or hypoxic condition + PTPRB-siRNA transfection for 36 h. *P < 0.05,**P < 0.01, ***P < 0.001

Hypoxia has a significant role in inducing EMT and metastasis in multiple cancers including CRC^17^. Hypoxia-inducible factor (HIF) is the key factor of physiological and pathological hypoxia response, which is significantly expressed in hypoxic environment. The qRT-PCR assay showed the level of HIF-1α was increased under hypoxia compared to the normoxia condition, indicating hypoxic condition was generated (Fig. 5a). After culture for 36 h in hypoxic conditions, CRC cells showed increased migration and invasion compared to control groups (Fig. 5b, c). However, PTPRB knockdown could reverse the effect of hypoxia on promoting migration and invasion. In addition, we had detected the effect of PTPRB on Twist under hypoxia. The twist expression was increased under hypoxia while PTPRB siRNA could reverse the effect of hypoxia on twist (Fig. S).

### PTPRB promotes tumor metastasis in vivo

**Fig. 6:**
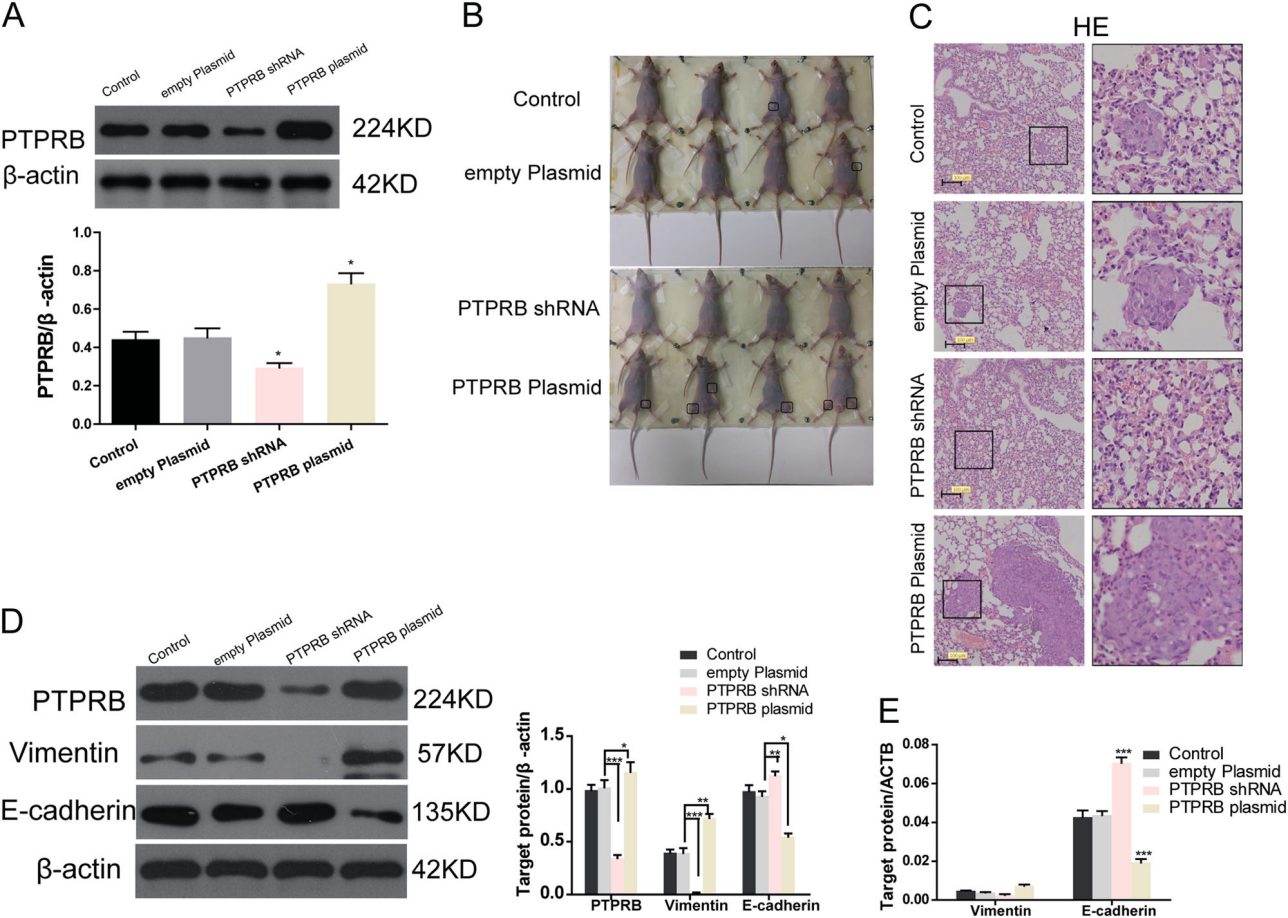
**a** The expression level of PTPRB in LOVO, HCT116, and HT29 cells after transfection of PTPRB-shRNA or PTPRB plasmid. **b**, **c** Metastasis in the whole body and lung in nude mice after injection of CRC cells transfected with PTPRB-shRNA or PTPRB plasmid. **d**, **e**Vimentin and E-cadherin expression in tumor tissues at protein level, *P < 0.05,**P < 0.01, ***P < 0.001 and transcript level, ***P < 0.001

To determine the effects of PTPRB expression level on tumor metastasis in vivo, PTPRB shRNA and PTPRB plasmids were transfected into CRC cells. Western blotting showed the transfection efficiency of PTPRB shRNA or PTPRB plasmid in HCT116 cells (Fig. 6a). Compared to mice injected with control cells, PTPRB knockdown cells inhibited tumor metastasis in the whole body (Fig. 6b), such as lung (Fig. 6c), while PTPRB overexpression increased tumor metastasis. Moreover, western blot analysis (Fig. 6d) and RT-PCR (Fig. 6e) revealed that PTPRB knockdown decreased vimentin expression and increased E-cadherin expression in tumor tissues. PTPRB overexpression showed an opposite effect on vimentin and E-cadherin expression.

### PTPRB is highly expressed in CRC

**Fig. 7:**
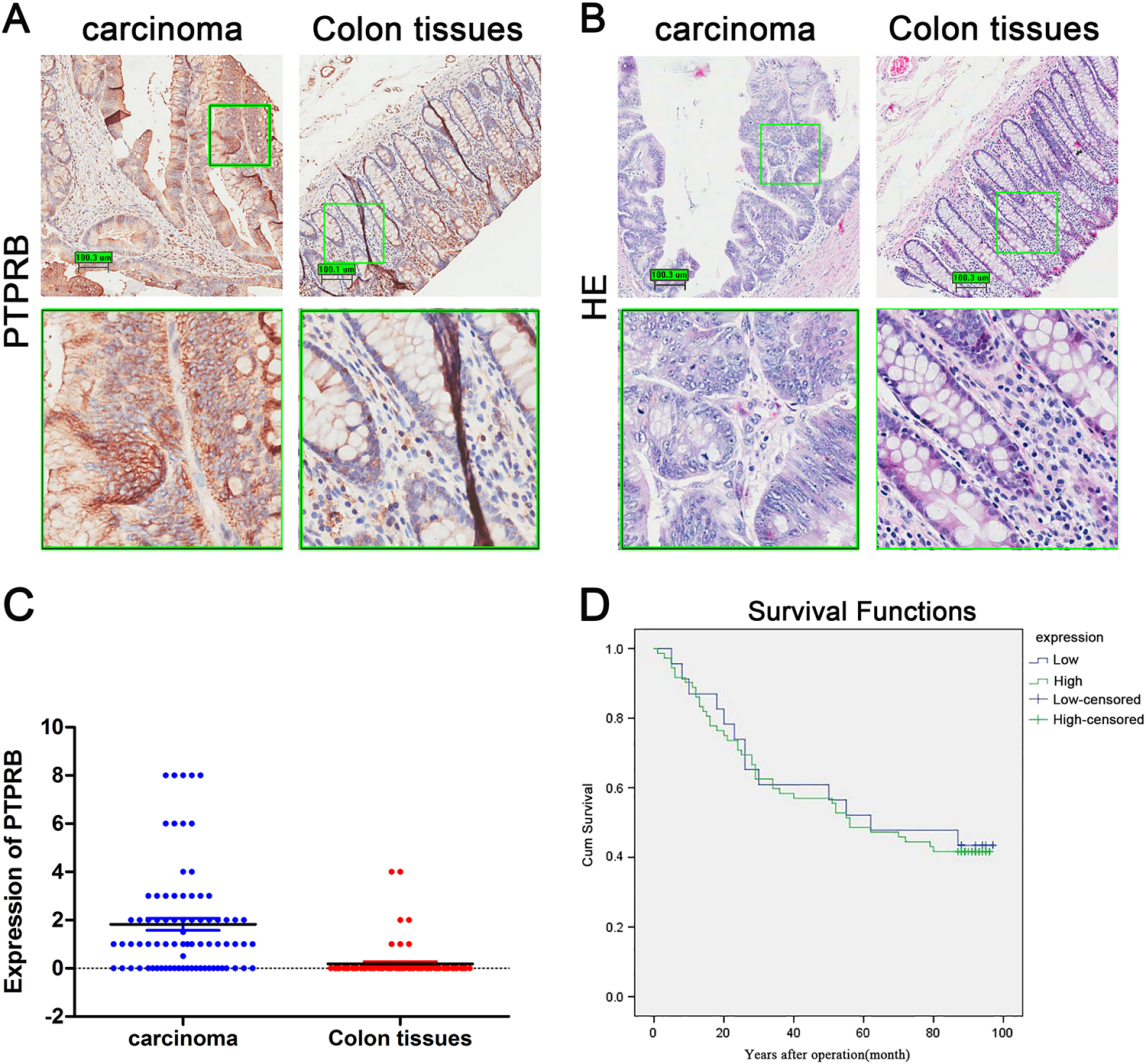
**a**The level of PTPRB expression in CRC and adjacent tissues was observed by histochemical stain. **b** HE staining of CRC tissues and adjacent nontumor tissues. **c** The expression level of PTPRB mRNA in CRC and adjacent tissues. **d** This study was used Kaplan–Meier survival analysis and log-rank statistical test to analyze the survival of PTPRB in 80 cancer tissues by single factor analysis

To analyze the expression of PTPRB in CRC, tumor tissues and adjacent nontumor tissues from 100 patients were assayed by immunohistochemistry and RT-PCR. As seen in Fig. 7a–c, the level of PTPRB mRNA and protein was significantly higher in CRC than in adjacent tissues. But as shown as in Fig. 7d, survival analysis indicated that there was no significantly difference between low expression and high expression of PTPRB.

## Discussion

PTPRB has been investigated in multiple primary malignancies and tumor cell lines^14,18,19^. However, there are few studies on the relationship between PTPRB function and CRC. This study firstly demonstrated that PTPRB is highly expressed in CRC tissues compared with adjacent nontumor tissues and in CRC cells with high metastasis potential. Metastasis is well known to be the main cause of CRC-related death, and more than 50% of patients with CRC will present with liver metastases during their lifespan^20^. PTPRB-siRNA significantly suppressed migration and invasion of CRC cells in vitro and inhibitory tumorigenesis in vivo. This result suggested that PTPRB was a potential molecular target against CRC metastasis. PTPRB could catalyze the dephosphorylation of phosphotyrosine residues, and along with protein tyrosine kinases could modulate the levels of phosphotyrosine modification in tumor cells.

Reversible phosphorylation of tyrosine accounts for less than 1% of the phosphoproteome, but it plays a disproportionately significant role in many diseases including cancer initiation and development^21^. Nearly 50% of the 90 human tyrosine kinases are implicated in cancer^22^, and many different RTKs are direct targets for protein tyrosine phosphatases^23–25^. Hence, PTPRB could regulate the proliferation, migration, invasion, and tumorigenesis of tumor cells as an essential regulator of the RTK signaling network.

Understanding the molecular mechanisms underlying how CRC cells acquire invasive and metastatic properties is essential for the development of effective strategies for CRC therapy. Increasing evidence supports the view that EMT could endow tumor cells with metastatic features via inducing loss of epithelial characteristics, for instance, cell depolarization, cell–cell disconnection, and transformation into an elongated, fibroblast-like morphology^26^. EMT is characterized by decreased expression of epithelial cell junction proteins such as E-cadherin, occludins, and claudins, and the upregulation of mesenchymal adhesion genes encoding, for instance, vimentin, fibronectin, and N-cadherin^27^. Among these, downregulation of E-cadherin expression results in the destabilization of adherens junctions and is a primary step for cancer metastasis. Therefore, we investigated the relationship between PTPRB and E-cadherin expression in CRC cells. Western blot analysis and immunofluorescence analysis showed that PTPRB overexpression induced higher vimentin expression and lower E-cadherin protein expression, while PTPRB knockdown resulted in an opposite effect on vimentin and E-cadherin protein expression in CRC cells. These results suggested that PTPRB plays a significant role in regulating EMT.

The regulation of epithelial and mesenchymal markers requires robust transcriptional machinery, consisting of three major groups of transcription factors: the TWIST family of basic helix-loop-helix transcription factors (TWIST1/TWIST2)^28^, the zinc finger E-box binding homeobox (ZEB) family of transcription factors (ZEB1/ZEB2)^29^, and the SNAIL family of zinc-finger transcription factors (SNAIL/SLUG)^30^. In this study, Twist1 siRNA was transfected into CRC cells to inhibit the EMT process. The results showed that Twist1 knockdown could eliminate the enhanced capacity for migration and invasion induced by PTPRB overexpression, suggesting that EMT may be the mechanism underlying the capacity for PTPRB to promote invasion.

In cancer cells, EMT is abnormally modulated by extracellular stimuli derived from the tumor microenvironment, for instance, inflammatory cytokines, growth factors, as well as intratumoral physical stresses such as hypoxia^24^. Therefore, we further cultured CRC cells in hypoxic conditions to induce EMT. We found that PTPRB knockdown inhibited expression of EMT markers even under hypoxic conditions. Altogether, these results provide compelling evidence supporting the prometastatic and pro-EMT function of PTPRB in CRC.

The in vitro experiments showed an antimetastasis role of PTPRB knockdown in CRC, and thus prompted us to evaluate its functions in tumor metastasis in vivo. The results showed that stable knockdown of PTPRB led to a significant inhibition of metastasis in lung and rectocolon in nude mice. Moreover, tumor sections from PTPRB knockdown xenografts showed lower levels of vimentin expression and upregulation of E-cadherin expression. Finally, the RT-PCR assay revealed that PTPRB was significantly upregulated in CRC tissues compared to adjacent nontumor tissues. Consistent with mRNA expression, immunohistochemical analysis showed that PTPRB was highly expressed in tumor tissues at the protein level. But, survival analysis showed that there was no significantly difference between low expression and high expression of PTPRB. It may be that the number of cases is not enough, or the meaning of cytoplasmic nuclear protein expression may be different. Anyway, these findings provide more comprehensive insight into the value of PTPRB as a potential CRC therapy target.

In conclusion, our study shows that PTPRB is upregulated in CRC. Functional data from both in vivo and in vitro assays strongly support the inference that PTPRB overexpression promotes the metastasis of CRC. Moreover, our study revealed the effect of PTPRB on promoting EMT, as reflected in increased vimentin and decreased E-cadherin expression potential, which provides a potential comprehensive mechanism by which PTPRB may promote metastasis.

## Materials and methods

### Clinical specimen collection and cell culture

Samples from 100 patients undergoing CRC resection were collected from Shanghai Outdo Biotech Company. Informed consent was obtained from all patients, and the experimental protocols agreed with local ethics committee regulations. Three CRC cell lines (LOVO, HCT116, and HT29) were purchased from Cell Bank of Type Culture Collection of Chinese Academy of Sciences, Shanghai Institute of Cell Biology, Chinese Academy of Sciences. LOVO and HT29 cells were cultured in 1640 complete medium supplemented with 10% fetal bovine serum (FBS) while HCT116 cells were cultured in McCoy’s 5A (Modified) Medium with 10% FBS at 37 °C in a humidified incubator with 5% CO_2_. Furthermore, the hypoxic condition in a hyposic incubator is 5%CO_2_, 94%N_2_, and 1%O_2_ at 37 °C.

### Transfection assay

The cells were seeded in a six-well plate at 1 × 105. When 80% confluence of cells, the siRNA (final concentration 100 nM, Ribobio, Guangzhou, China) or plasmid (2 μg, Hanbio, Shanghai, China) was transfected into the cells with liposome lipo 2000 (Invitrogen, California, USA). The lipo was diluted (5 μl/well) and the siRNA or plasmid was diluted at the calculated concentration by OPTIM-MEM (Gibco Company, Massachusetts, USA), respectively, and then the two were mixed in a proportional manner for 15 min. The cells were transfected by the mixed medium in an incubator for 6–8 h and then cultured in a normal culture medium. For in vivo study, the siRNA proved effective in vitro was packaged into lentivirus (Hanbio, Shanghai, China). In order to generate stable transfected cell lines, puromycin and G418 were separately used to screen for lentivirus and plasmid transfected cells. The survival cells were collected and cultured. Western Blot was used to detect the transfection efficiency. The siRNA sequences targeting PTPRB and Twist 1 are listed as follows:

Twist 1,

Twist1-homo-1575 5′ GGUGUCUAAAUGCAUUCAUTT 3′

5′ AUGAAUGCAUUUAGACACCTT 3′

Twist1-homo-810 5′ GGUACAUCGACUUCCUCUATT 3′

5′ UAGAGGAAGUCGAUGUACCTT 3′

Twist1-homo-780 5′ GCAAGAUUCAGACCCUCAATT 3′

5′ UUGAGGGUCUGAAUCUUGCTT 3′

PTPRB,

PTPRB-Homo-1817(human) 5′ GCAGAACAUUUCCAGACAATT 3′

5′ UUGUCUGGAAAUGUUCUGCTT 3′ (For lentivirus package)

PTPRB-Homo-2364(human) 5′ CCAAGUGACUGACUUGCAUTT 3′

5′ AUGCAAGUCAGUCACUUGGTT 3′

PTPRB-Homo-3685(human) 5′ GCAUCUGUCCAAGGAGUAATT 3′

5′ UUACUCCUUGGACAGAUGCTT 3′

### Total RNA extraction and RT-PCR

Total RNA from cells and CRC tissues were extracted using a TRIzol Total RNA extraction Kit (Invitrogen Co.), and reverse-transcribed to cDNA using a TaqMan Reverse Transcription Kit (Applied Biosystems). Quantitative real-time PCR (qRT-PCR) analysis was performed using a Takara SYBR Premix Ex Taq system (Applied Biosystems). All experiments were performed in triplicate. The nucleotide sequences of the primers used for qRT-PCR (Shanghai Sangon Biological Engineering Technology Services Co., Ltd) are as follows:

PTPRB,

Ptprb-F 5′ CACAGAGATGCAATCTACTCGAGAC 3′

Ptprb-R 5′ CAACAGAAATGGCTGGCACC 3′

Actin,

Actin-F 5′ TGGCACCCAGCACAATGAA 3′

Actin-R 5′ CTAAGTCATAGTCCGCCTAGAAGCA 3′

Vimentin,

VIM-F 5′ TGAGTACCGGAGACAGGTGCAG 3′

VIM-R 5′ TAGCAGCTTCAACGGCAAAGTTC 3′

E-cadherin,

E-cadherin-F 5′ TACACTGCCCAGGAGCCAGA 3′

E-cadherin-R 5′ TGGCACCAGTGTCCGGATTA 3′

### Western blotting analysis

Protein from cells and tissues were lysed using RIPA buffer (Beyotime, Jiangsu, China) supplemented with protease inhibitors PMSF (Beyotime). Total protein concentration was determined by the BCA assay. In all, 20–50 μg total protein was subjected to electrophoretic separation in SDS-PAGE gel analysis, and then transferred to PVDF membranes at 350 mA for 90 min. Subsequently, the membranes were blocked using 5% non-fat milk in TBS supplemented with 0.1% Tween-20 for 1–2 h. After washing three times, the membranes were incubated with primary antibodies overnight at 4 °C, and further incubated with an appropriate secondary antibody for 2 h. The expression levels of target proteins were visualized using enhanced chemiluminescence.

Primary antibodies against PTPRB (1:1000), Vimentin (1:1000), E-cadherin (1:1000), Goat antiRabbit HRP antibody, and Goat antiMouse HRP antibody were purchased from Cell Signaling Technology (Beverly, MA, USA).

### Tissue microarray and Immunohistochemistry

The expression level of PTPRB in tissue was determined by tissue microarray (TMA). TMAs were assembled by manual tissue punch. Different samples were drilled from selected tissue areas and assembled into new paraffin blocks. The histological cores are 2 mm in diameter and 4–6 mm in length. The immunohistochemistry stainings were performed on 4 μm sections. The sections were deparaffinized and rehydrated, then subjected to heat-induced epitope retrieval. The activity of endogenous peroxidase was quenched with 3% hydrogen peroxide, and the sections were then blocked using 5% FBS in TBST. After washing three times, the primary monoclonal antibody was added and sections were incubated overnight at 4 °C. After incubation with HRP-conjugated secondary antibodies for 1 h at room temperature, protein expression was visualized using 3,3′-diaminobenzidine with hematoxylin stain for contrast.

the standardization of the original experimental data:

1. Staining intensity score: 0 points (negative), 1 point (1+), 2 points (2+), 3 points (3+);
2. Staining positive rate score: 0 points (negative), 1 point (1–25%), 2 points (26–50%), 3 points (51–75%), 4 points (76—100%);
3. Total score and grouping: The product of “staining intensity score” and “staining positive rate score” is grouped into total scores; ≤2 is divided into antibody low expression group, >2 is divided into antibody high expression group. (depending on the distribution of data).

### Wound healing assay

A total of 3 × 10^5^ CRC cells were seeded in six-well plates containing 2 ml culture medium. After achieving 80–90% confluence, an open wound was scratched into the cell monolayer using 200 μl pipettes and the medium was replaced with serum-free medium. The capacity of cell migration was determined as the percentage of recovery following a 36 h incubation period.

### Transwell invasion experiment

A total of 1 × 105 CRC cells in 200 μl serum-free 1640 medium were distributed into the upper chambers of each well (24-well insert; 8-mm pore size; Millipore, Billerica, MA, USA) coated with Matrigel (BD Bioscience). Culture medium supplemented with 10% FBS was added in the lower chambers as a chemoattractant. After incubation for 24 h, cells remaining in the upper chambers were wiped off, and the cells on the reverse aspect of the Matrigel membrane were fixed with 4% paraformaldehyde for 20 min. The number of invasive cells was recorded after staining with 0.4% crystal violet.

### Animal studies

BALB/c-nu male nude mice (age: 4–5 weeks; weight: 20–25 g) (Experimental Animal Center of Zhejiang University, Hangzhou, Zhejiang, China) were grouped into Control, empty plasmid, shRNA-PTPRB (lentivirus), and PTPRB plasmid groups, with at least eight mice in each group and approved by the Second Affiliated Zhejiang Hospital, Zhejiang University of Medical Ethics Committee and the Medical Faculty Ethics Committee of the Second Affiliated Zhejiang Hospital, Zhejiang University. All experiments were performed in accordance with the Guide for the Care and Use of Experimental Animals guidelines and regulations. HCT116 cells or stable tranfected cells at the logarithmic phase of growth were collected, counted and suspected by physiological saline solution. Nude mice were injected with 0.2 ml cell suspension (1.0 × 10^7^ cells/ml) via tail vein. Six weeks later, took photograph. Next, lung tissue was isolated, fixed in 10% formalin for HE staining.

### Statistical analysis

All experimental data are presented as mean with SD. The difference between two groups was analyzed using unpaired Student’s *t*-test. Survival analysis curves were plotted using the Kaplan–Meier method. *P* < 0.05 was considered to be statistically significant. Statistical analysis was performed using SPSS 17.0 software (SPSS).

## Supplementary information

supplement material

Supplementary Figure
