# Supplementary material for: PTPRB promotes metastasis of colorectal carcinoma via inducing epithelial-mesenchymal transition
Source: Cell Death Dis. 2019 Apr 30;10(5):352. doi: 10.1038/s41419-019-1554-9 (PMC6491493; doi:10.1038/s41419-019-1554-9)
Supplement: Supplementary file 1 — supplement material [file 41419_2019_1554_MOESM1_ESM.docx]

Figure S (A-B). Twist expression in LOVO, HCT116, and HT29 cells transfected with PTPRB-siRNA or PTPRB plasmid was analyzed by Western blotting analysis. (C) Twist expression in LOVO, HCT116, and HT29 cells under hypoxia was analyzed by Western blotting analysis, *P < 0.05,** P < 0.01, *** P < 0.001.
