# Supplementary figures and images for: PTPRB promotes metastasis of colorectal carcinoma via inducing epithelial-mesenchymal transition
Source: Cell Death Dis. 2019 Apr 30;10(5):352. doi: 10.1038/s41419-019-1554-9 (PMC6491493; doi:10.1038/s41419-019-1554-9)

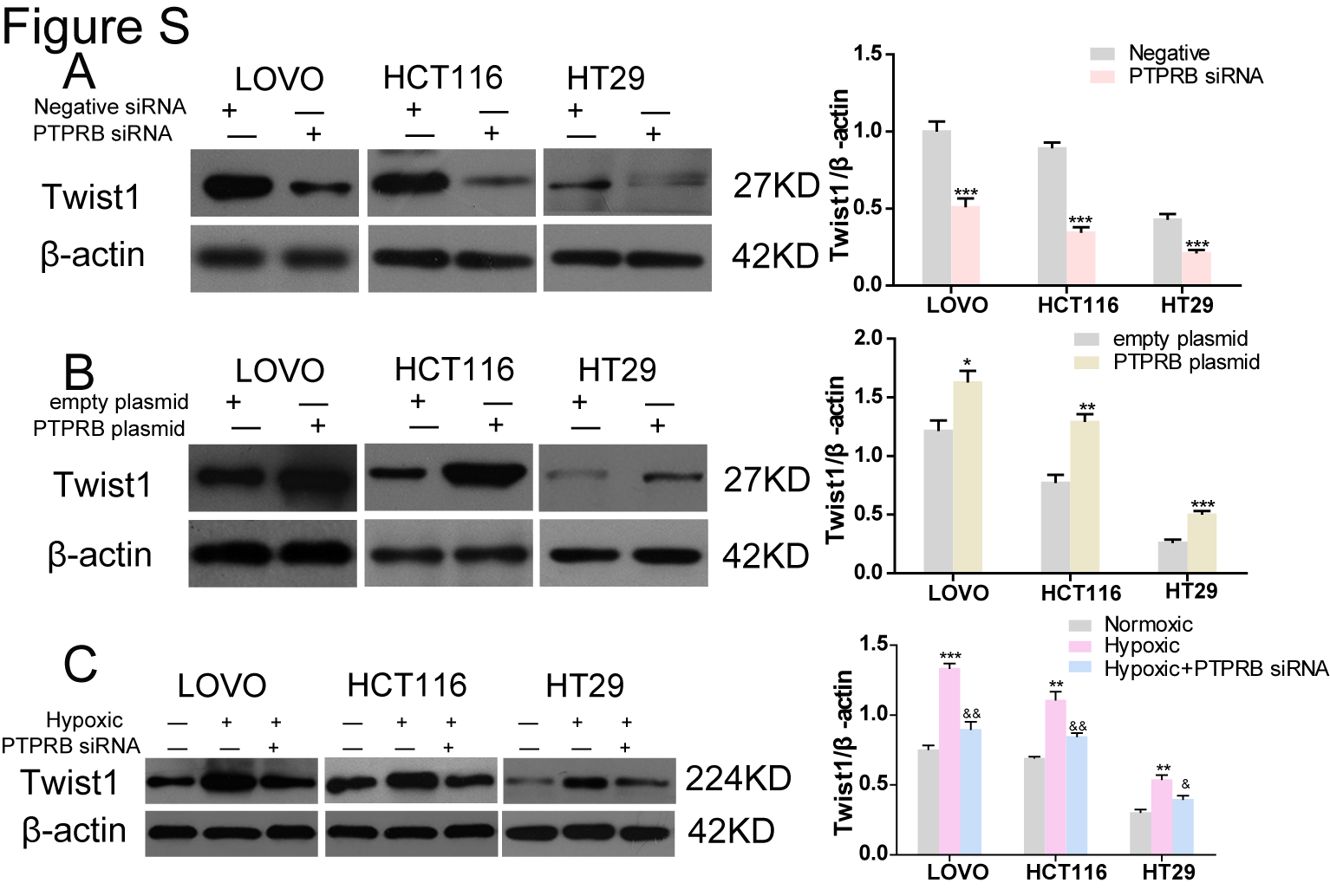

Supplement: Supplementary file 2 — Supplementary Figure [file 41419_2019_1554_MOESM2_ESM.tif]
